# Auditory Encoding of Natural Speech at Subcortical and Cortical Levels Is Not Indicative of Cognitive Decline

**DOI:** 10.1523/ENEURO.0545-23.2024

**Published:** 2024-05-08

**Authors:** Elena Bolt, Nathalie Giroud

**Affiliations:** ^1^Computational Neuroscience of Speech and Hearing, Department of Computational Linguistics, University of Zurich, Zurich 8050, Switzerland; ^2^International Max Planck Research School on the Life Course (IMPRS LIFE), University of Zurich, Zurich 8050, Switzerland; ^3^Language & Medicine Centre Zurich, Competence Centre of Medical Faculty and Faculty of Arts and Sciences, University of Zurich, Zurich 8050, Switzerland

## Abstract

More and more patients worldwide are diagnosed with dementia, which emphasizes the urgent need for early detection markers. In this study, we built on the auditory hypersensitivity theory of a previous study—which postulated that responses to auditory input in the subcortex as well as cortex are enhanced in cognitive decline—and examined auditory encoding of natural continuous speech at both neural levels for its indicative potential for cognitive decline. We recruited study participants aged 60 years and older, who were divided into two groups based on the Montreal Cognitive Assessment, one group with low scores (*n* = 19, participants with signs of cognitive decline) and a control group (*n* = 25). Participants completed an audiometric assessment and then we recorded their electroencephalography while they listened to an audiobook and click sounds. We derived temporal response functions and evoked potentials from the data and examined response amplitudes for their potential to predict cognitive decline, controlling for hearing ability and age. Contrary to our expectations, no evidence of auditory hypersensitivity was observed in participants with signs of cognitive decline; response amplitudes were comparable in both cognitive groups. Moreover, the combination of response amplitudes showed no predictive value for cognitive decline. These results challenge the proposed hypothesis and emphasize the need for further research to identify reliable auditory markers for the early detection of cognitive decline.

## Significance Statement

Research on cognitive decline needs more studies uncovering markers for subtle neural changes in the pre-dementia stage. Neural markers for auditory processing have a potential that has not been widely explored in studies. Here, for the first time, we used natural, continuously spoken language to examine neural processing in two groups of older adults with and without cognitive decline. We quantified how well the brain tracks speech not only at the cortical but also at the subcortical level. In contrast to previous research suggesting that subcortical and cortical auditory responses are enhanced in cognitive decline, we found no group differences. We believe that this study represents an important contribution to the search for markers of cognitive health in old age.

## Introduction

Considering the increasing prevalence of dementia worldwide, markers for early detection are of crucial importance. Dementia is preceded by a protracted, preclinical phase before symptoms in cognition and behavior manifest. By the time they become apparent, the disease is often advanced and brain damage is irreversible. Therefore, more subtle neuropathological changes that precede cognitive decline are of particular interest. A promising approach for early detection lies in the neurophysiology of auditory processing (Bidelman et al., 2017). When a sound reaches the ear, it undergoes complex sensory and cognitive processing before meaning can be derived. This pathway involves subcortical relay stations in the brainstem and midbrain and processing stations in the cortex (Ades and Brookhart, 1950). Cognition and hearing are closely linked (Lindenberger and Baltes, 1994) and age-related hearing loss is associated with cognitive decline (Lin and Albert, 2014; Wayne and Johnsrude, 2015; Thomson et al., 2017). Despite this link, studies investigating neurophysiological auditory processing in dementia are scarce and often neglect auditory function. Hearing loss affects auditory processing, with the most evident effects manifesting as reduced and delayed encoding in the brainstem (Verhulst et al., 2016; Presacco et al., 2019), leading to increased cortical representation (Decruy et al., 2020; Fuglsang et al., 2020).

There is one study that suggested a link between cognitive decline and auditory encoding hypersensitivity. Bidelman et al. (2017) showed that adults with signs of cognitive decline exhibited increased activity at the brainstem level, which was subsequently reflected in increased cortical representation. They used electroencephalography (EEG) to examine frequency-following responses (FFRs) and auditory evoked potentials (AEPs). The FFR serves as indicator of the auditory system’s ability to encode fundamental frequency and harmonics (Skoe and Kraus, 2010) and originates from subcortical structures influenced by the brainstem and the inferior colliculus in the midbrain (Chandrasekaran and Kraus, 2010). The AEP represents a cortical response to auditory stimuli and has three peaks: a positive deflection (P1) approximately 50 ms after stimulus onset, followed by a negative deflection (N1) at 100 ms and a positive deflection (P2) at 200 ms (Picton and Hink, 1974). P1 and N1 refer to initial detection, while P2 reflects higher level processing of information. Bidelman et al. (2017) reported that adults with signs of cognitive decline showed increased FFR-residual mean squared (RMS) amplitudes and N1–P2 voltage differences, providing a model for predicting decline based on response amplitudes. Despite the promising implications of these findings, it is notable that no subsequent research has yet validated and built on these observations.

Recent methodological advances in neurophysiological auditory processing allow the experimental use of natural continuous speech. Such stimuli have a stronger real-world connection than repetitive sounds, enhancing the ecological validity of results and making experiments more engaging for participants. Speech is characterized by low-frequency envelope modulations. During listening, the cortex synchronizes its activity with the temporal structure of the envelope, essentially “tracking” it (Luo and Poeppel, 2007). To quantitatively assess this speech tracking, linearized stimulus-response models known as temporal response functions (TRFs) are used (Crosse et al., 2016). TRF models are created by regressing a speech-derived feature against the EEG, resulting in a time-lagged model that describes the relationship between the two signals. These models exhibit deflections akin to P1–N1–P2 complexes and allow neurophysiological interpretation (Crosse et al., 2021). TRF models have recently been extended to subcortical processing (Maddox and Lee, 2018; Bachmann et al., 2021; Kulasingham et al., 2024), which show a peak approximating wave V in auditory brainstem responses (ABRs), reflecting activity in subcortical structures and occurring approximately 5 ms after sound onset. The advantage of TRF models over conventional potentials lies in their ability to assess the response to speech over a longer listening period.

Building on this framework, we investigated neurophysiological speech processing in participants with and without signs of cognitive decline. We used an auditory EEG paradigm inspired by Bidelman et al. (2017) and Bachmann et al. (2021), which allowed us to simultaneously record subcortical and cortical responses while participants listened to an audiobook, and complemented it with an ABR recording. To control for hearing ability, we performed audiometric tests to ensure that the observed differences were due to cognitive factors and not hearing impairment. Our study was guided by two hypotheses: First, that cognitive decline is associated with encoding hypersensitivity at cortical and subcortical levels, as evidenced by increased TRF and evoked responses. Second, we expected that response peak amplitudes might serve as predictors of cognitive decline, providing novel insight into the neurophysiological markers of cognitive health in older adults.

## Materials and Methods

### Participants

We invited 45 participants, aged 60 and older, to take part in our study. Recruitment was conducted through an advertisement with Pro Senectute (prosenectute.ch). All participants were native Swiss German speakers, raised monolingually until the age of seven years, right-handed, and retired. Our selection process involved a preliminary telephone interview, followed by a detailed on-site interview for final participants. We confirmed right-handedness using the Annett Hand Preference Questionnaire (Annett, 1970). Exclusion criteria were set to avoid confounding factors such as a professional music career, dyslexia, significant neurological impairments including dementia, depression, severe or asymmetrical hearing loss (defined as average pure-tone hearing threshold exceeding 60 dB hearing level (HL) or an interaural difference exceeding 20 dB HL), and the use of hearing aids. Eventually, we had to exclude one participant due to technical issues. This study was conducted with the approval of the Ethics Committee of the Canton of Zurich (BASEC No. 2019-01400). All participants provided written informed consent and received monetary compensation for their participation. A study session lasted approximately three and a half hours and was conducted at the Linguistic Research Infrastructure (LiRI, liri.uzh.ch).

#### Montreal Cognitive Assessment-based cognitive grouping

**Figure 1.**
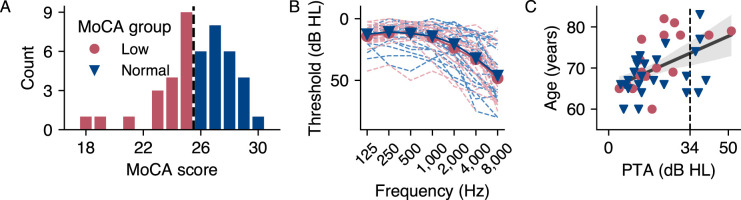
Overview over Montreal Cognitive Assessment (MoCA) scores and audiometric results by MoCA group and age. ***A***, Distribution of MoCA scores. The dashed line indicates the clinical threshold for mild cognitive impairment (MCI). Participants scoring below the threshold of 26 points were assigned to the low MoCA group, and participants greater or equal to the threshold were assigned to the normal MoCA group. ***B***, Individual hearing thresholds for each frequency averaged by ear. ***C***, Four-frequency pure tone average (PTA) thresholds plotted against age. The dashed line indicates the threshold of 34 dB HL, above which hearing impairment is considered moderate. PTA and age were significantly correlated across all participants. The shaded error bar represents the 95% confidence interval.

In line with Bidelman et al. (2017), we grouped participants based on cognitive performance as assessed by the Montreal Cognitive Assessment (MoCA; Nasreddine et al., 2005). All experiment instructors had completed training certification prior to data collection (see mocacognition.com/training-certification). MoCA scores range from 0 to 30 points, where higher scores indicate better cognitive function. Using a cutoff score of 26 points, the clinical threshold for mild cognitive impairment (MCI), participants were divided into two groups: Participants with signs of cognitive decline (low MoCA group, 19 participants with 23.6 ± 2.1 points; 12 women, 7 men) and participants with normal cognitive performance (normal MoCA group, 25 participants with 27.4 ±1.2 points, 14 women, 11 men). The distribution of MoCA scores across groups is depicted in Figure 1*A*. Age for the low MoCA group was 71.7 ± 6.3 (range: 60–82) years and for the normal MoCA group, 68.6 ± 5.3 (range: 60–83) years. The age difference between the groups was not statistically significant (two-tailed Mann–Whitney *U* test: *U* = 311.5, *p* = 0.081, *r* = 0.31).

### Audiometric testing

We conducted audiometric testing using the Affinity Compact audiometer (Interacoustics, Middelfart, Denmark), equipped with a DD450 circumaural headset (Radioear, New Eagle, PA, USA). Participants sat in a soundproof booth to minimize external interference during the assessments.

#### Pure-tone audiometry

Pure-tone hearing thresholds were measured for both ears at frequencies of 125, 250, 500, 1,000, 2,000, 4,000, and 8,000 Hz, with individual thresholds averaged by ear presented in Figure 1*B*. We based the overall hearing ability on the four-frequency pure-tone average (PTA), calculated as the average of thresholds at 500, 1,000, 2,000, and 4,000 Hz (Lin and Reed, 2021). The interaural PTA difference was 4.5 ± 4.4 (range: 0–17.5) dB HL, indicating symmetrical hearing. PTA was then averaged across both ears, with the low MoCA group averaging 20.4 ± 11.5 dB HL and the normal MoCA group averaging 19.6 ± 11.8 dB HL. Most participants had no to mild hearing impairment, although seven had moderate impairment, i.e., a PTA exceeding 34 dB HL (Humes, 2019), two of them in the low MoCA group and five in the normal MoCA group. No significant difference in hearing ability was observed between groups (Mann–Whitney *U* test: *U* = 262.5, *p* = 0.561, *r* = 0.11). However, PTA correlated with age (Fig. 1*C*; *ρ* = 0.5, *p* = 0.0003, Fisher’s *z* = 0.57, *n* = 44).

#### Speech-in-noise

To complement pure-tone audiometry, we conducted a speech-in-noise (SiN) test, which is more cognitively demanding and relevant for understanding cognitive decline (Moore et al., 2014). Using the SiN test provided by the Affinity software, participants repeated two-syllable compound words embedded in white noise (fixed at 65 dB HL), with the speaker’s level adjusted for comprehension. We determined the SiN perception level as the signal-to-noise ratio (SNR) at which participants correctly repeated a word at least twice out of three repetitions. SNR loss values, higher values indicating poorer perception, were averaged for both ears. The low MoCA group had an average SNR loss of 5.5 ± 2.5 (range: 2–12) dB HL, and the normal MoCA group 5.9 ± 2.5 (range: 2–14) dB HL, within the normal to mild loss range (Taylor, 2003). We observed no significant difference in SNR loss between groups (Mann–Whitney *U* test: *U* = 216.5, *p* = 0.612, *r* = −0.09), and SNR loss was not age-related (*ρ* = −0.1, *p* = 0.497, Fisher’s *z* = 0.11, *n* = 44).

PTA and SNR loss were correlated (*ρ* = 0.5, *p* = 0.0003, Fisher’s *z* = 0.57, *n* = 44), indicating that the two measures of hearing ability lead to a comparable result. Finally, we chose PTA as a control variable for hearing ability in further analyses.

### EEG recording

We recorded EEG using the Biosemi ActiveTwo system (Biosemi, Amsterdam, The Netherlands) with 32 electrodes (10–20 system), four external electrodes and at a sampling rate of 16.384 kHz. We positioned two external electrodes above and below the right eye to measure the electrooculogram (EOG), and two on the left and right mastoids. Throughout the experiment, we monitored and maintained electrode offsets below 20 μV. Participants sat in a soundproof, electromagnetically shielded booth for the recordings. During the listening tasks, we instructed them to focus on a fixation cross displayed on a screen and to minimize movement, especially when the cross was visible. The entire EEG recording session, including preparation and breaks, lasted for approximately 90 min. This session comprised four parts: an initial click-ABR measurement, a resting-state measurement, and two speech-related paradigms using audiobook segments and matrix sentences, respectively. The resting state and matrix sentences paradigms are not discussed in this paper.

### Audio playback and experiment control

To display instructions and play auditory stimuli to participants, we used the Presentation® software (Neurobehavioral Systems, Inc., Berkeley, CA, USA; research resource identifier (RRID): SCR_002521). However, we opted for audio file triggering to achieve the level of temporal precision required for neurophysiological experiments concerned with subcortical processing. Specifically, we encoded the onset of auditory inputs into the stereo wav container. The first channel contained the auditory stimulus—speech and click trains, respectively—while the second channel held pulses coinciding with the analysis-relevant sound onsets in the stimulus. The first channel was connected to the audio system, and the second channel was routed to the EEG system. This integration eliminated the latency between the playback of the stimulus on the sound card and the input of the port code into the EEG system. Speech and click trains were presented bilaterally through electromagnetically shielded ER3 insert earphones (Etymotic Research, Elk Grove Village, IL, USA). The sound card used was a RME Fireface UFX II (RME Audio, Haimhausen, Germany). To validate the timing accuracy, we measured the latency between the input of the trigger into the EEG system and the output at the earphones using an artificial ear. This resulted in a constant latency of 1.07 ms, which we accounted for in the analysis.

### Click stimuli

Following the procedure and technique outlined by Bachmann et al. (2021), we conducted click-ABR recordings. The click-ABR measurements involved presenting a train of rectangular-shaped clicks, each lasting 80 μs, with alternating polarities, and a repetition rate of 10 Hz. Each participant listened to 3,000 click repetitions, which amounted to a total duration of 5 min. For consistent presentation, we employed the peak equivalent sound pressure level (peSPL) calibration method (Laukli and Burkard, 2015) to set the sound level. Clicks were presented at 93 dB peSPL.

### Natural speech stimuli and task

We used excerpts from Sylvia Plath’s novel, “The Bell Jar”, identical to those used by Kurthen et al. (2021). Participants listened to 25 segments from the German audiobook version, which featured a professional female speaker with a fundamental frequency (*F*0) of 160.5 ± 8.9 Hz. The recording was sampled at 44.1 kHz and was organized into content-coherent segments. We used Praat (Boersma and Van Heuven, 2001, RRID: SCR_016564) to limit silent gaps (defined by a threshold ≤−20dB dB) to 500 ms and to scale the average speech wave intensity to 70 dB SPL. Each segment commenced with a short silence of approximately 467 (range: 330–500) ms, which we retained to introduce stimulus onset jitter. The average final duration of each segment was 45.7 ± 2.7 s, resulting in a total listening time of approximately 20 min. We calibrated the sound level such that segments were consistently played at 70 dB peSPL.

To sustain participants’ attention, we interspersed the listening sessions with simple comprehension questions related to the content they were hearing. The first question was asked after the first segment to familiarize participants with the task. The remaining questions were asked after every third section, so we analyzed a total of eight questions (excluding the first one) to assess participants’ attention. These questions followed a forced-choice format, where participants selected one of four answer choices by pressing a key.

### Data preprocessing

Our auditory EEG paradigm enabled the derivation of both a subcortical and a cortical TRF from the same speech and EEG data through different preprocessing steps. Crucial to this paradigm are the high sampling rate and the use of trigger pulses with the highest temporal precision we could achieve with our equipment. To supplement the TRF analyses, we also computed click-ABR and AEP derived from the speech onsets. We performed the data preprocessing using Python (version 3.11.6) and the MNE-Python package (Gramfort et al., 2013, RRID: SCR_005972). Before processing the speech waves, we removed the silent periods before the trigger pulses so that time zero of the speech wave matches time zero in the EEG signal. Before processing the EEG, we examined the raw data to identify bad electrodes, with an average of 1.5 ± 1.6 bad electrodes per participant. After their removal, the EEG signals were referenced to the linked mastoids, with the exception of six participants with noisy mastoid electrodes, where the cap electrodes T7 and T8 were used (Bachmann et al., 2021). We processed speech and EEG signals with equivalent filters whenever possible, and for the subcortical analyses, causal filters were used in the data preprocessing pipeline whenever feasible (Maddox and Lee, 2018; Bachmann et al., 2021). Anti-alias filters were consistently applied at a cutoff frequency of 1/3 of the target rate.

#### Subcortical data

Our protocol for the subcortical processing pipeline was largely based on Maddox and Lee (2018), Bachmann et al. (2021), and Kulasingham et al. (2024). We extracted speech features from audiobook segments using the auditory nerve firing rate model by Zilany et al. (2014). This model, based on inner ear physiology, simulates auditory nerve responses to acoustic stimuli and has proven effective in subcortical TRF modeling, outperforming other speech-derived features (Kulasingham et al., 2024; Shan et al., 2024). We adapted the implementation by Shan et al. (2024) with the Python cochlear package (Rudnicki et al., 2015). The process involved upsampling the speech wave to 100 kHz and converting it to a pascal pressure waveform at 70 dB SPL. We then simulated the firing rates of 43 auditory nerve fibers across logarithmically spaced center frequencies from 125 Hz to 16 kHz. The model outputs, representing the nerve rate response, were averaged to produce a single time series, which was then downsampled back to 44.1 kHz. This procedure was performed for both the original and inverted speech waves, following methodologies described by Maddox and Lee (2018), Shan et al. (2024), and Kulasingham et al. (2024).

In alignment with clinical ABR settings and conventions in subcortical TRF modeling (Bachmann et al., 2021; Kulasingham et al., 2024), we used the EEG signal from the vertex electrode Cz. For three participants with noisy Cz electrodes, we used Pz instead as vertex electrode, as previously done by Bachmann et al. (2020). We subjected speech features and EEG to the following preprocessing steps: to eliminate power line noise, we applied infinite impulse response (IIR) notch filters at all multiples of 50 between 50 and 1,350 Hz to the continuous EEG (zero-phase non-causal Butterworth filter, effective order: 8, all transition bandwidths: 5 Hz). We applied an anti-alias filter to the speech features at 1,365.3 Hz using a one-pass, non-linear phase, causal low-pass finite impulse response (FIR) filter with a Hamming window (0.0194 pass-band ripple, 53 dB stop-band attenuation, upper transition bandwidth: 409.6 Hz, filter length: 357 samples), after which the speech features were resampled to the target rate of 4,096 Hz using the resample function of MNE. The EEG data were filtered from 80 to 1,365.3 Hz using a one-pass, non-linear phase, causal band-pass FIR filter with a Hamming window (0.0194 pass-band ripple, 53 dB stop-band attenuation, lower transition bandwidth: 20 Hz, upper transition bandwidth: 409.6 Hz, filter length: 2,705 samples). We chose the additional lower limit of 80 Hz to reduce cortical contributions (Bidelman et al., 2017; Bachmann et al., 2021). Continuous EEG data were segmented into epochs from −4 to 54 s, in which time point zero corresponds to the onset of speech, and then decimated to 4,096 Hz. For minimal EEG cleaning, we zeroed out 1-s segments around samples exceeding 100 μV, following an approach comparable to Maddox and Lee (2018) and Kulasingham et al. (2024). Initially, we replaced the bad segments with NaNs, which would then be replaced with zeros after feature-target scaling described in “TRF modeling”. Finally, we truncated the first and last second of each speech feature, and cropped the EEG epochs to 1–49 s relative to speech onset, which corresponds to the length of the longest speech segment post-truncation. We performed this step to avoid fitting the model to the neural response elicited by the onset of the sound and to discard possible filtering artifacts (Crosse et al., 2021).

#### Cortical data

A recent study by Lindboom et al. (2023) showed that model responses of the inferior colliculus, such as auditory nerve responses, also improve the performance of cortical stimulus-response models compared to, for example, the envelope or spectrogram of a speech stimulus. Inspired by their work, we decided to also use the nerve rate response derived from the original speech wave as speech features for cortical TRF modeling. All filters applied in the cortical processing pipeline were zero-phase, non-causal FIR filters with a Hamming window (0.0194 pass-band ripple, 53 dB stop-band attenuation) with varying pass-band frequencies and transition bandwidths. We subjected the speech features and EEG to the following preprocessing steps: before downsampling, we applied an anti-alias filter at 170.7 Hz (upper transition bandwidth: 51.2 Hz, filter length: 775 samples for the speech features, 1,057 for the EEG due to the difference in sampling rates) to the speech features and the continuous EEG. We segmented the continuous EEG data into epochs from −4 to 54 s relative to speech onset, and then decimated them to 512 Hz, while the speech features were resampled to 512 Hz. We created a copy of the epochs instance for independent component analysis (ICA) and high-pass filtered the epochs copy at 1 Hz (lower transition bandwidth: 1 Hz, filter length: 1,691 samples), a process which facilitates ICA decomposition (Klug and Gramann, 2020). We employed the Picard algorithm (Ablin et al., 2018) to perform ICA on the epochs copy with 1,000 iterations, with the goal to retain 99.9% of the signal’s variance. We further enhanced ICA performance using five iterations within the FastICA algorithm (Hyvarinen, 1999). After ICA fitting, we automatically labeled components associated with eye-related artifacts with the EOG external electrodes as references, and manually labeled components associated with muscle activity or singular artifacts based on topography, temporal occurrence, and frequency spectrum. On average, we excluded 2.5 ± 0.8 components per participant. We zeroed out the components in the original epochs instance and then performed electrode interpolation. We further downsampled speech features and EEG signals to 128 Hz, using an anti-alias filter at 42.7 Hz (lower transition bandwidth: 12.8 Hz, filter length: 133 samples), which was followed by decimation for the EEG and resampling for the speech features. Finally, we band-pass filtered the EEG and speech features at 1–9 Hz (lower transition bandwidth: 1.0 Hz, upper transition bandwidth: 2.25 Hz, filter length: 423 samples). In line with the subcortical data, we truncated the first and last second of each speech feature and cropped the EEG epochs to 1–49 s relative to speech onset.

Within the same preprocessing pipeline, we also obtained AEP responses to the audiobook segment onsets. To this end, we copied the epochs after the final filtering step, cropped them to −300 to 600 ms relative to speech onset, applied a baseline correction from −200 to −50 ms, and then averaged the signals across epochs to obtain the evoked response to 25 speech onsets.

#### Click-ABR data

The EEG signal from the click-ABR recording was subjected to the same subcortical filtering procedure as the EEG from the speech experiment, with the analysis performed on vertex electrode Cz and with the same filter specifications. After filtering, we segmented the EEG into epochs from −10 to 30 ms and applied a baseline correction from −10 to −5 ms. We retained the vertex electrode signals and excluded epochs that exceeded 40 μV, which lead to an average exclusion of 117.8 ± 222.6 epochs per participant. We then averaged the signal over epochs to obtain the ABR response.

### TRF modeling

#### Matrix representation of feature-target pairs

For TRF modeling, as required by the ReceptiveField (see “TRF modeling”), we stored speech features and EEG epochs in matrix format. Since the audiobook segments varied in length and we wanted to avoid data loss, we decided to zero-pad shorter segments up to the longest segment length instead of taking an approach where we truncate longer segments to a uniform length corresponding to the shortest segment. We thus padded shorter segments with NaN to reach the length of the longest segment, which was 48 s after preprocessing. The NaN were replaced with zeros after feature-target scaling as described in “Modeling procedure”. We have stored the speech features and the EEG in matrices, which can be represented as $F\in R^{e\times c\times t}$ and $R\in R^{e\times c\times t}$, where *e* denotes the number of epochs, *c* the number of channels, and *t* the number of time samples. The speech features and the subcortical EEG matrices each comprised one channel (the speech feature and the EEG signal from the vertex electrode), while the cortical EEG matrices included 32 channels.

#### Modeling procedure

To quantify speech-evoked subcortical and cortical responses, we used forward TRF models (Crosse et al., 2016). We built the models using the ReceptiveField class by MNE and cross-validation tools by Scikit-Learn (Pedregosa et al., 2011, RRID: SCR_002577). These models are based on a time-lagged linearized ridge regression with a Laplacian regularization term that imposes a smoothing condition for nearby time samples. The deconvolution equation for the TRF model weights vector **w** is given by

(1) $$w=(S^{⊤}S+\lambda M{)}^{-1}S^{⊤}r,$$

where **S** is the time-delayed matrix of a single-segment speech feature, **r** is the corresponding EEG response, *λ* is the regularization parameter, and **M** is the Laplacian matrix (for matrix definition, see Crosse et al., 2016, p. 3). The weights vector **w** is found by minimizing the squared mean error between the predicted EEG response $\hat{r}$ and the actual EEG response **r**.

Participant-specific tuning involved randomly splitting data into training and test sets, for which we used 22 segments for training and three for testing. For feature-target scaling, we z-standardized the EEG responses globally before the split and normalized speech features using training set statistics. After this scaling step, we replaced all NaNs in the padded speech features and to be zeroed out subcortical EEG 1-s segments with zeros. We opted for this approach such that the scaling procedure would not be affected by the zero-padding and zeroing-out of the data. We determined the optimal *λ* by performing a grid search over *λ* = 10^*n*^, with *n* = { − 3, − 2, …, 9} using leave-one-out cross-validation. Specifically, each of the 22 segments in the training set was used as a validation segment once. Pearson’s correlation coefficients between predicted and actual EEG responses served as encoding accuracies. We averaged the encoding accuracies across all cross-validation folds and chose the *λ* that maximized the mean encoding accuracy. A final model was fitted on the training set with the optimal *λ* and then tested for encoding accuracy on the held-out test set, which did not contribute to the final TRF model. Subcortical TRF models were fitted for time lags between −10 and 30 ms, cortical models spanned −300 to 600 ms. We fitted the subcortical model twice (for both the original and inverted speech features) and averaged the weights and encoding accuracies for a unified subcortical TRF model. This procedure has been shown to reduce the influence of stimulus artifacts, similar to the common alternating polarity technique used in click-ABR (Maddox and Lee, 2018; Bachmann et al., 2023; Kulasingham et al., 2024; Shan et al., 2024). To assess the encoding accuracies of the cortical models, we averaged the encoding accuracies across all electrodes.

### Code accessibility and data availability

The Python scripts for data preprocessing described in “Data preprocessing” and for TRF modeling described in “TRF modeling” are available at github.com/elbolt/subCortex-speech. The data used in this study are available upon request from the corresponding author.

### Cluster-based response peak extraction

**Figure 2.**
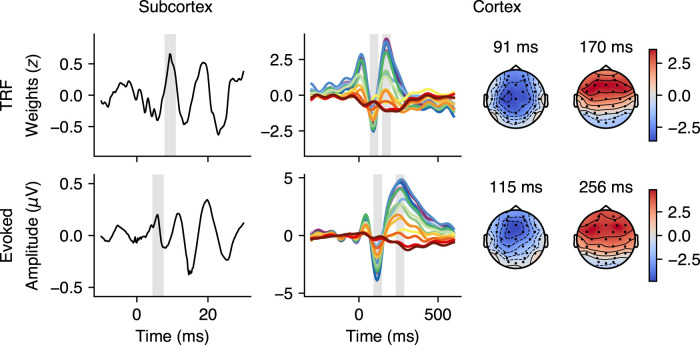
Global average of temporal response functions (TRFs) and evoked response waveforms across participants with topographical maps at peak latencies. The top panel shows the global subcortical TRF at vertex electrode Cz, with peak at 9.3 ms interpreted as a wave V-like (left) and global cortical TRF at all electrodes, with P1–N1–P2-like complex, N1 peak at 91 ms and P2 peak at 170 ms (right). The bottom panel shows the global click-auditory brainstem response (ABR) with wave V peak at 5.8 ms (left) and global auditory evoked response (AEP) with N1 peak at 115 ms and P2 peak at 256 ms (right). The gray shaded areas around the peaks of interest represent a 3 ms window around the subcortical peaks and a 50 ms window around the cortical peaks. Topographical maps extracted from the 50 ms windows around the peak latencies show that the cortical TRF and AEP were particularly pronounced at frontotemporal electrodes.

We extracted response peaks from subcortical and cortical TRF weights, click-ABR, and AEP. For subcortical analyses, we focused on wave V, while for cortical analyses, we concentrated on N1 and P2 peaks. We applied baseline corrections to the TRF weights by subtracting the mean weights between −10 and −5 ms for subcortical models and −200 and −50 ms for cortical models. These weights were then z-standardized across all participants. Click-ABR and AEP data remained in μV units. We initially examined the global TRF and evoked response waveforms averaged across all participants. Figure 2 shows the neural components we obtained from our data, with the TRF models in the top half and the evoked responses in the bottom half. The global subcortical TRF at vertex electrode Cz showed a peak at 9.3 ms, resembling wave V. The global cortical TRF response across electrodes exhibited a P1–N1–P2-like complex, with an average N1 peak at 91 ms and P2 peak at 170 ms, most pronounced at frontotemporal electrodes. The lower half of Figure 2 displays the global click-ABR and AEP waveforms. The click-ABR identified a peak at 5.8 ms as wave V. The global AEP showed N1 and P2 peaks at 115 ms and 256 ms, respectively, with a frontotemporal distribution similar to the cortical TRF.

#### Electrode clustering

Before performing the analysis in an electrode cluster, we investigated whether there were electrode-based cortical processing differences between the groups. To this end, we determined the mean amplitude within a 50 ms window around the grand average N1 and P2 peaks (shown in Fig. 2) for each participant and each electrode and calculated permutation tests (Maris and Oostenveld, 2007) to compare the mean amplitudes between the groups. Since this analysis did not reveal any significant clusters (not shown), we decided to base our peak extraction on an auditory-relevant frontotemporal cluster, consisting of electrodes F3, FC1, FC5, FC6, FC2, and F4, as in previous studies (Di Liberto et al., 2015; Hjortkjær et al., 2020; Bachmann et al., 2021; Bachmann, 2022).

#### Peak extraction

For each participant, we identified a subcortical wave V-like peak between 4 and 11 ms and extracted its mean amplitude using a 3 ms window centered on the peak. For cortical responses, we identified N1-like peaks within 50–120 ms and P2-like peaks within 120–300 ms in the mean response of the frontotemporal cluster and extracted the mean amplitudes within a 50 ms window around these peaks. The click-ABR peak was similarly extracted between 4 and 9 ms, while the AEP N1 and P2 peaks were extracted within 50–150 ms and 150–300 ms windows, respectively.

### Statistical analyses

We performed all statistical analyses, including the statistical parameters reported in the Materials and Methods section, using JASP (version 0.18.3; RRID: SCR_015823). Given the limited sample size and the deviation from normality of the data residuals, we used non-parametric tests. In general, we analyzed the data using frequentist approaches, but supplemented the testing of our two hypotheses with an analytical Bayesian approach. This combination ensures robustness under non-normal conditions and allows a nuanced interpretation of the parameter uncertainties. We calculated Spearman’s rank correlation coefficient when examining correlations and Mann–Whitney *U* tests to compare variables between MoCA groups.

In testing our first hypothesis that peak amplitudes are increased with cognitive decline, we performed an additional Bayesian Mann–Whitney *U* test to obtain a Bayes factor (BF) for each peak amplitude, assuming a Cauchy prior width of *γ* = 0.707, the default setting in JASP, and a prior that is considered weakly informative (Kruschke, 2015). In addition to the BF, we evaluated the region of practical equivalence (ROPE) to assess the evidence for the absence of an effect (Kruschke, 2018). We defined the ROPE bounds as ±0.1, aligning with Cohen’s criteria for a small effect size. Using a custom Python script, we determined the proportion of the posterior distributions—represented by the 95% credible interval (CrI)—that fell within the ROPE range. This proportion is reported as the ROPE percentage.

In testing our second hypothesis that peak amplitudes predict signs of cognitive decline, we implemented generalized linear models assuming a Bernoulli distribution and a corresponding logit link function equivalent to logistic regression. Building on the approach of Bidelman et al. (2017), we used the absolute difference between the N1 and P2 peak amplitudes as a predictor in this model. Predictors thus included wave V amplitude, N1–P2 amplitude difference, and their interaction. Additionally, we controlled for PTA and age. The model was configured with the following formula:

(2) MoCA group∼wave V amplitude×N1−P2 amplitude difference+PTA+age.

Additionally, we performed a Bayesian logistic regression using the same model configuration. We set a uniform prior for all predictors to have a noninformative baseline. The Markov chain Monte Carlo method was used to draw 5,000 samples and estimate the posterior distributions of the model parameters, yielding a BF and posterior distributions for each predictor. Again, we calculated the ROPE for each predictor to determine the evidence for the absence of an effect. We performed the logistic regression analyses twice, once for TRF-derived and once for click-ABR- and AEP-derived peak amplitudes.

## Results

### Behavioral performance in audiobook task

Overall, participants performed well on the audiobook questions. In the low MoCA group, participants answered 92±9% of the questions correctly with a reaction time of 8.3 ± 2.4 (range: 5.9–14.6) s, and participants in the normal MoCA group answered 93±9% correctly, with a reaction time of 7.9 ± 2.4 (range: 5–15.4) s. Both groups performed equally well on question accuracy (two-tailed Mann–Whitney *U* test: *U* = 221, *p* = 0.673, *r* = −0.07), and had comparable reaction times (*U* = 274, *p* = 0.398, *r* = 0.15). No participant scored less than 75% on the task (range in both groups: 75– 100%), i.e., all participants answered at least six out of eight questions correctly. The purpose of the questions was to maintain attention during the recording, and from these results we concluded that participants listened attentively to the audiobook and that we could consider their EEG data for further analysis.

### Encoding accuracies

**Figure 3.**
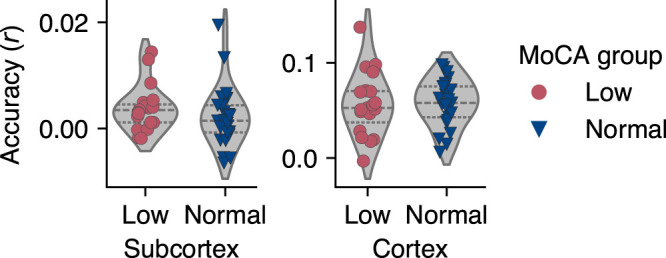
Encoding accuracies (Pearson’s correlation coefficients) of the temporal response functions (TRFs) for subcortical (left) and cortical (right) models, plotted by Montreal Cognitive Assessment (MoCA) group.

We first examined whether the encoding accuracies of the TRF models differed between the groups. This step was important to ensure that any observed differences in response peaks were not primarily confounded by fluctuations in encoding accuracy. The subcortical models showed lower accuracies than the cortical models, but these were comparable to those reported by Bachmann (2022) for participants in a similar age range. Subcortical and cortical encoding accuracies did not differ between groups (two-tailed Mann–Whitney *U* test: *U* = 287, *p* = 0.248, *r* = 0.21, and *U* = 223, *p* = 0.743, *r* = −0.06, respectively). Figure 3 shows the subcortical and cortical encoding accuracies plotted by group. As some participants’ subcortical models scored particularly low on the test set, we examined the correlations between subcortical and cortical encoding accuracy to determine whether low accuracy at the subcortical level was also associated with low accuracy at the cortical level. In the low MoCA group, subcortical and cortical accuracies were not associated (*ρ* = 0.2, *p* = 0.240, Fisher’s *z* = 0.17, *n* = 19). In the normal MoCA group, accuracies at both levels were also not significantly related (*ρ* = −0.3, *p* = 0.943, Fisher’s *z* = −0.34, *n* = 25). These results suggested that the encoding accuracies were not associated with cognitive grouping.

### TRF models

#### Response latencies and amplitudes between groups

**Figure 4.**
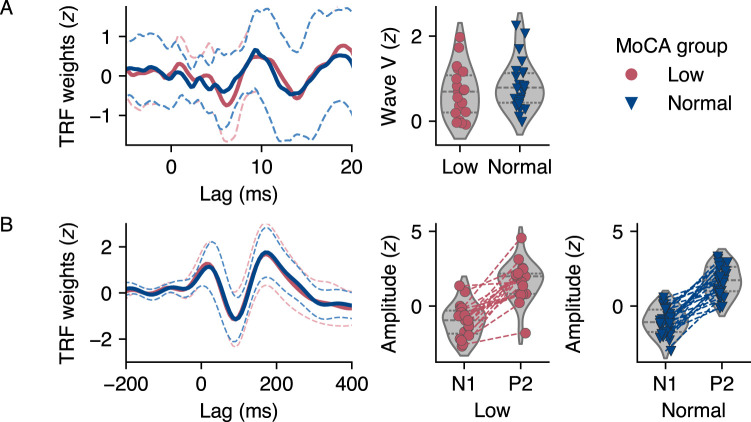
Mean temporal response function (TRF) model weights and peak amplitudes by Montreal Cognitive Assessment (MoCA) group. The dashed line around the response represents the standard deviation of the mean. ***A***, Subcortical TRF at vertex electrode Cz (left) and individual wave V-like amplitudes (right). ***B***, Cortical TRF averaged over frontotemporal cluster consisting of electrodes F3, FC1, FC5, FC6, FC2, F4 (left), and individual N1 and P2-like amplitudes, connected by a line (right).

The subcortical TRF models showed visual variability in the time-lagged response weights. We identified wave V-like peaks at a latency of 8.2 ± 2.5 ms in the low MoCA group and at 8.5 ± 2.2 ms in the normal MoCA group (range in both groups: 4–11 ms). Peak latency was not different between groups (two-tailed Mann–Whitney *U* test: *U* = 228, *p* = 0.830, *r* = −0.04). The average response weights by group and the amplitudes of the individual wave V-like peaks are shown in Figure 4*A*. In contrast, the cortical TRF models showed a more consistent pattern of response weights. We identified an N1-like peak at a latency of 89 ± 11 (range: 76–115) ms in the low MoCA group and at 90 ± 15 (range: 52–115) ms in the normal MoCA group. The P2-like peak occurred at 175 ± 19 (range: 146–217) ms in the low MoCA group and at 181 ± 22 (range: 154–248) ms in the normal MoCA group. Neither N1 nor P2 peak latencies differed significantly between groups (two-tailed Mann–Whitney *U* test: *U* = 219.5, *p* = 0.672, *r* = −0.08, and *U* = 203.5, *p* = 0.422, *r* = −0.14, respectively). The average response weights by group and the individual N1- and P2-like peak amplitudes are shown in Figure 4*B*.

We then tested whether the amplitudes of the response peaks were increased in participants with signs of cognitive decline. Contrary to our expectation, amplitudes were not increased in the low MoCA group, neither for the wave V-like peak (one-tailed Mann–Whitney *U* test: *U* = 199, *p* = 0.820, *r* = −0.16) nor for the N1- and P2-like peaks (*U* = 243, *p* = 0.453, *r* = 0.02, and *U* = 232, *p* = 0.463, *r* = 0.02, respectively). Bayesian Mann–Whitney *U* tests confirmed these results, showing moderate evidence for the null hypothesis with a BF of 0.16 ( 95% CrI: 0.01–0.443) for the wave V-like peak, and anecdotal evidence for the null hypothesis with a BF of 0.33 ( 95% CrI: 0.01–0.66) for the N1 peak and 0.30 ( 95% CrI: 0.01–0.62) for the P2 peak. ROPE analyses indicated that 21.7%, 14.1%, and 15.1% of the posterior distributions of effect sizes fell within ranges considered practically insignificant for the wave V-like, N1, and P2 peaks, respectively, suggesting that any observed effects were not of substantial size. These percentages indicate that, while there is some overlap with the no-effect range, the majority of the effect size estimates for each peak point toward an effect that is not negligible, albeit not substantial in size. Overall, our analyses do not support the hypothesis that signs of cognitive decline are associated with increased subcortical and cortical activation during encoding, as the observed effects, if present, appear not to be of substantial magnitude.

#### Predictive potential of TRF weights for group

The TRF-based logistic regression results are summarized in Table 1. Contrary to our expectations neither wave V amplitude (*z* = −0.66, *p* = 0.664), N1–P2 difference (*z* = −0.34, *p* = 0.458) nor their interaction (*z* = 0.39, *p* = 0.440) emerged as significant predictors for the MoCA group in the frequentist framework. Control variables PTA (*z* = 0.95, *p* = 0.340) and age (*z* = −1.90, *p* = 0.058) also showed no significant effects. The Bayesian analysis supported these findings, yielding a BF of 0.55 for wave V amplitude, 0.48 for N1–P2 difference, and 0.48 for their interaction, all indicating anecdotal evidence for the null hypothesis. For the control variables with a BF of 0.64 and 1.41, respectively, the evidence for the null hypothesis was also anecdotal for PTA and anecdotal against the null hypothesis for age. In the ROPE analysis, 11.2% of the posterior distribution for the wave V amplitude, 31.8% for N1–P2 difference, and 48.8% for their interaction were within ranges considered negligible in terms of effect size. Similarly, 100% and 75% of the posterior distributions for PTA and age fell within the trivial effect range. These results collectively suggest a lack of substantial predictive value in the combination of subcortical and cortical response peak amplitudes.

**Table 1.** Logistic regression coefficients for TRF response peaks, PTA, and age on MoCA group

	Frequentist						Bayesian				
					95% CI					95% CrI	
	Estimate	*SE*	*z*	*p*	LL	UL	BF	*M*	*SD*	LL	UL
Intercept	10.11	5.47	1.85	0.065	0.12	22.02	1.00	4.46	5.02	−1.93	14.45
Wave V (*z*)	−0.66	1.52	−0.43	0.664	−3.79	2.34	0.55	0.06	0.40	−0.74	1.03
N1–P2 difference (*z*)	−0.34	0.46	−0.74	0.458	−1.31	0.54	0.48	−0.01	0.13	−0.35	0.28
PTA (dB HL)	0.03	0.04	0.95	0.340	−0.03	0.11	0.64	0.01	0.02	−0.03	0.04
Age (years)	−0.14	0.08	−1.90	0.058	−0.31	−0.01	1.41	−0.03	0.05	−0.14	0.02
Wave V × N1–P2	0.39	0.50	0.77	0.440	−0.56	1.47	0.48	0.01	0.10	−0.11	0.30

*Note.* Montreal Cognitive Assessment (MoCA) group low is coded as class 0, MoCA group normal as class 1. TRF, temporal response function; PTA, pure-tone average; SE, standard error; CI, confidence interval; LL, lower limit; UL, upper limit; BF, Bayes factor; M, mean; SD, standard deviation; CrI, credible interval.

### Evoked responses

#### Response latencies and amplitudes between groups

**Figure 5.**
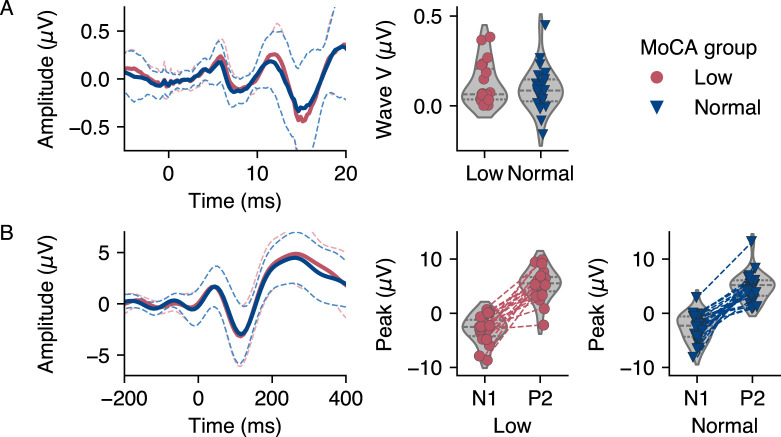
Mean evoked responses and peak amplitudes by Montreal Cognitive Assessment (MoCA) group. The dashed line around the response represents the standard deviation of the mean. ***A***, Click-auditory brainstem responses (ABRs) at vertex electrode Cz (left) and individual wave V amplitudes (right). ***B***, Auditory evoked potentials (AEPs) averaged over frontotemporal cluster consisting of electrodes F3, FC1, FC5, FC6, FC2, F4 (left), and individual N1 and P2 amplitudes, connected by a line (right).

In the individual click-ABR waveforms, we identified peaks of wave V with a latency of 5.7 ± 1.2 (range: 4–8.8) ms in the low MoCA group and at 5.5 ± 1.0 (range: 4–8.6) ms in the normal MoCA group. Latencies between groups did not differ significantly (two-tailed Mann–Whitney *U* test: *U* = 267, *p* = 0.241, *r* = 0.13). Although the AEP was based on the speech onsets of only 25 audiobook segments, all participants showed individual waveforms that allowed unambiguous identification of N1 and P2 peaks. We identified N1 peaks at a latency of 112 ± 20 (range: 52–138) ms in the low MoCA group and at 119 ± 10 (range: 91–138) ms in the normal MoCA group. The P2 peaks occurred at 252 ± 31 (range: 201–295) ms in the low MoCA group and at 251 ± 33 (range: 185–295) ms in the normal MoCA group. Neither N1 nor P2 latencies differed between groups (two-tailed Mann–Whitney *U* test: *U* = 185.5, *p* = 0.898, *r* = −0.22, and *U* = 238.5, *p* = 0.495, *r* = 0.01, respectively). The peaks of evoked responses by group and the individual peak amplitudes are shown in Figure 5.

 Concerning the amplitudes of the response peaks, the same pattern emerged as in the TRF analysis. In the frequentist framework, the amplitudes were not increased in the group with low MoCA. This was observed for the wave V peak (one-tailed Mann–Whitney *U* test: *U* = 256, *p* = 0.336, *r* = 0.08), and similarly for the N1 and P2 peaks (*U* = 224, *p* = 0. 629, *r* = −0.06, and *U* = 273, *p* = 0.205, *r* = 0.15). The Bayesian tests confirmed these results. For the wave V peak, the BF was 0.51 ( 95% CrI: 0.02–0.75), with 11.4% of the posterior distribution within the ROPE. This indicates anecdotal evidence for the null hypothesis, suggesting that the effect might not be substantial. For the N1 peak, a BF of 0.25 ( 95% CrI: 0.01–0.57) and 16.3% of the effect size distribution within the ROPE imply stronger support for the null hypothesis, indicating a negligible effect. Lastly, the P2 peak, with a BF of 0.49 ( 95% CrI: 0.01–0.74) and 12.1% of the effect size distribution within the ROPE, showed anecdotal evidence for the null hypothesis.

#### Predictive potential of evoked response peaks for group

The results of the evoked response-based logistic regression analysis are summarized in Table 2. In alignment with the TRF-based analysis, the frequentist approach revealed that the main effects and interaction did not predict MoCA group, neither for wave V amplitude (*z* = 0.22, *p* = 0.825), nor N1–P2 difference (*z* = −0.36, *p* = 0.716), and their interaction (*z* = −0.58, *p* = 0.563). Similarly, the control variables, PTA (*z* = 0.61, *p* = 0.542) and age (*z* = −1.81, *p* = 0.071), were also not significant. The Bayesian analysis suggested anecdotal evidence against the inclusion of these predictor with a BF of 0.61 for wave V amplitude, 0.69 for the N1–P2 difference, and 0.63 for their interaction, indicating marginal support for the null hypothesis. In the ROPE analysis, 2.31% of the posterior distribution for wave V amplitude, 76% for the N1–P2 difference, and 25% for the interaction term fell within ranges considered negligible in terms of practical significance. Again, for the control variables, there was anecdotal evidence against the inclusion of PTA, with a BF of 0.65, and anecdotal evidence in favor of the inclusion of age, with a BF of 1.69. This interpretation is substantiated by the ROPE analysis, where 100% of the posterior distribution for PTA and 70.1% for age were within the ROPE, suggesting that a substantial proportion of the effect sizes are practically equivalent to zero.

**Table 2.** Logistic regression coefficients for ABR and AEP response peaks, PTA, and age on MoCA group

	Frequentist						Bayesian				
					95% CI					95% CrI	
	Estimate	*SE*	*z*	*p*	LL	UL	BF	*M*	*SD*	LL	UL
Intercept	10.25	5.39	1.90	0.057	0.55	22.22	1.00	5.75	5.51	−1.28	16.63
Wave V (μV)	1.30	5.90	0.22	0.825	−10.42	14.68	0.61	−0.34	1.85	−4.83	3.83
N1–P2 difference (μV)	−0.05	0.14	−0.36	0.716	−0.32	0.23	0.69	−0.02	0.06	−0.16	0.09
PTA (dB HL)	0.02	0.04	0.61	0.542	−0.05	0.10	0.65	0.01	0.02	−0.03	0.04
Age (years)	−0.14	0.08	−1.81	0.071	−0.31	−52.82	1.69	−0.04	0.05	−0.15	0.02
Wave V × N1–P2	−0.43	0.75	−0.58	0.563	−2.09	0.96	0.63	−0.02	0.18	−0.48	0.32

*Note.* Montreal Cognitive Assessment (MoCA) group low is coded as class 0 and MoCA group normal as class 1. ABR, auditory brainstem response; AEP, auditory evoked potential; PTA, pure-tone average; SE, standard error; CI, confidence interval; LL, lower limit; UL, upper limit; BF, Bayes factor; M, mean; SD, standard deviation; CrI, credible interval.

## Discussion

The main objective of this study was to investigate the neurophysiological processing of natural continuous speech in older adults with and without signs of cognitive decline at both the subcortical and cortical levels. To this end, we used a novel paradigm based on TRF models that allowed us to examine speech encoding at both levels from the same EEG recording. Contrary to our first hypothesis, we did not observe increased TRF response amplitudes in the group with signs of cognitive decline, neither at the subcortical nor at the cortical level. This result is consistent with our analyses of the ABR to click sounds and the AEP to audiobook onsets. Additionally, response peak amplitudes from subcortical and cortical sources did not reveal any predictive value for cognitive decline, leading us to refute our second hypothesis. Thus, our results did not support the proposed hypothesis of auditory encoding hypersensitivity in the early stages of cognitive decline and could not confirm their predicative value for cognitive decline. Instead, auditory response patterns appeared to be similar in both groups of participants. While the Bayesian approach validated the frequentist analysis, it is important to note that, first, the evidence for the null hypotheses was only anecdotal and, second, the use of noninformative priors represents a conservative stance that may limit sensitivity to the detection of subtle but significant effects.

Our outcomes contrast with the findings of Bidelman et al. (2017), who found that older adults with signs of cognitive decline had increased FFR amplitudes in response to syllable sounds, which was then reflected in increased N1 and P2 AEP amplitudes at the cortical level. In their study, they trained a linear discriminant analysis classifier (Fisher, 1936) to discriminate participants with and without evidence of cognitive decline based on the RMS amplitude in the 50–150 ms window of FFR and N1–P2 AEP voltage difference. They were able to classify participants into the respective groups with a cross-validated accuracy of 80%, with FFR RMS being the stronger predictor of cognitive decline. This finding was particularly striking because, although the MoCA is a screening tool and does not provide a clinical diagnosis, the results by Bidelman et al. (2017) suggested that the pathophysiological effects of cognitive decline may extend to auditory processing by the time an individual exhibits a low score on the MoCA test. Our study did not aim to replicate the work of Bidelman et al. (2017), but rather to confirm and extend the results using a different paradigm aimed primarily at using natural speech instead of syllable sounds. The use of natural speech not only better corresponds to real-life listening experiences but also allows for a broader understanding of auditory processing. And another big advantage of this approach is that subcortical and cortical responses can be estimated from the same data and do not require separate paradigms, which enhances its clinical applicability. This approach overcomes some of the limitations of repetitive experimental stimuli by capturing the complexity and variability of everyday speech, providing a better context for studying auditory and cognitive functions.

### Components of auditory processing as predictors of cognitive decline

We hypothesized that TRF models could capture a more comprehensive component of cognitive components in natural speech processing than evoked potentials. This is attributed to the fact that TRF models incorporate the entire temporal sequence of stimulus and response, unlike evoked potentials which average responses over a brief period following sound onset. The subcortical auditory activity is influenced both by bottom-up input signals and top-down corticofugal connections to the auditory cortex (Chandrasekaran et al., 2014). Considering the hypersensitivity hypothesis in cognitive decline postulated by the findings of Bidelman et al. (2017), we anticipated similar or more pronounced effects in our TRF models, given the enhanced interplay between predictive top-down modulation of neural dynamics and bottom-up sensing in speech tracking (Zion Golumbic et al., 2012). The conclusions we drew from the TRF models bear limitations, particularly regarding the subcortical models, as discussed in “Methodological challenges of the subcortical TRF analysis”. Nevertheless, the analyses of evoked potentials confirmed the conclusions from the TRF models and are also not consistent with the hypersensitivity hypothesis. We would like to note that “hypersensitivity” within the scope of our study refers only to the increased subcortical and cortical responses as observed by Bidelman et al. (2017) in individuals with signs of cognitive decline, and not to the cortical hyperactivity in auditory processing as discussed mainly in the context of hearing loss (Herrmann and Butler, 2021, for an overview).

Focusing on subcortical processing, it is important to clarify that the click-ABR is not directly analogous to the FFR: we targeted wave V, a key component of subcortical auditory processing. We chose this focus because subcortical TRF models’ wave V-like peaks closely resemble the click-ABR wave V (Maddox and Lee, 2018; Bachmann et al., 2021), primarily originating from subcortical sources. The inferior colliculus in the midbrain is identified as the main generator of wave V (Møller and Jannetta, 1983). A simulation study by Saiz-Alía and Reichenbach (2020) suggested that the subcortical response to natural speech is also predominantly influenced by the inferior colliculus. In contrast, the FFR is influenced by both subcortical and cortical activities (Coffey et al., 2016, 2019), suggesting that cortical processing differences, reflected in N1–P2 complexes, might interact with FFR variations. Our results suggest that neither the amplitudes of the subcortical TRF nor the click-ABR waves are indicative of early cognitive decline. It might be that the FFR has a higher predictive potential for signs of cognitive decline after all. Compared to the click-ABR, which is used clinically primarily to assess auditory neuropathy and hearing loss, the FFR can detect other deficits in auditory processing that have more real-life consequences (Krizman and Kraus, 2019).

At the cortical level, our findings correspond with the conclusions drawn in the review by Morrison et al. (2018) on the use of AEP components as markers of cognitive decline: they reported that the N1 and P2 components did not consistently differ between patients with MCI, Alzheimer’s disease, and healthy controls. Rather, the review proposed that mismatch negativity (MMN)-related N200 and P300 components derived from active and passive oddball paradigms be examined as potential indicators of sensitivity to auditory differentiation and auditory attention, respectively. MMN-related components have also been associated with age-related differences in cortical thickness and surface area (Giroud et al., 2019), and may prove to be more sensitive to cognitive decline than the N1 and P2 components.

Looking ahead, further research is required to identify the most sensitive auditory processing components for cognitive decline. While we modeled acoustic speech aspects through auditory nerve firing rates, TRF models can also incorporate linguistic features derived from the speech signal such as words and phoneme rates and even higher order linguistic processing such as surprisal, entropies, and frequencies at the word or phoneme level (Brodbeck et al., 2018; Weissbart et al., 2020; Gillis et al., 2022, 2023; Brodbeck et al., 2023). The potential of such models has already been explored, for example in differential speech processing in patients with aphasia after a stroke (Kries et al., 2023). There is also potential in the MMN-related components, against the background of which we would like to make an argument for the relevance of the stimulus material. In the scope of the predictive coding theory in the auditory cortex (Heilbron and Chait, 2018), natural speech, particularly when supplemented with audiovisual elements like the speaker’s face, as well as context manipulation, could be beneficial. This is supported by Chauvin et al. (2021), who found that patients with MCI’s speech-in-noise comprehension benefited significantly when context was enhanced in sentences or when audiovisual speech cues were available, compared to healthy participants. Future research should therefore aim to identify the most effective auditory processing components for detecting cognitive decline, encompassing acoustic, linguistic, and cognitive aspects of speech processing.

### Relationship between auditory processing and cognitive decline

While our results did not suggest a link between auditory processing and cognition in the measures we studied, this does not necessarily mean that there is no connection between the two. It is plausible that this link might be more pronounced under adverse listening conditions or during complex linguistic processing or speech-in-noise tasks, as suggested by models like the ease of language understanding model (Rönnberg et al., 2013). This perspective assumes that auditory–cognitive connections may be more prominent in demanding listening environments or in tasks that require higher linguistic processing. Consequently, in a generally healthy group of older individuals, such as our participants, these connections may only manifest in more demanding scenarios. This hypothesis is consistent with the potential importance of components such as the MMN for auditory differentiation and attention (Morrison et al., 2018; Giroud et al., 2019). A promising direction for future research would therefore be to investigate this relationship under more demanding listening conditions. The inclusion of this aspect could lead to a more differentiated understanding of the interplay between auditory processing and cognitive decline.

### Participant selection and effects of the MoCA score on cognitive grouping

The selection of participants was a critical factor in our study. We speculate that individuals who chose to participate are likely to represent rather high-functioning members of their age group, especially in research on cognition and hearing loss in old age, despite some of them achieving a low MoCA score. This selection bias affects the generalizability of our results. Factors such as participants’ motivation to participate in cognitive studies, their general health status, and their cognitive resilience play a central role.

In general, the use of the MoCA as a criterion for assigning participants to different cognitive groups poses challenges. While the MoCA is an effective, internationally used screening tool for the detection of potential cognitive decline, it is of course not equivalent to a comprehensive clinical diagnosis. In addition, some participants in our sample were around the 26 threshold. Nine participants scored 25, placing them in the lower MoCA group, while six participants scored 26, placing them in the normal group. Re-running the statistical pipeline excluding participants with scores 25 and 26, the results did, however, not change. The same was true for the exclusion of three participants who had a score below 22 (Fig. 1*A*). In addition, we exploratively examined regression models that treated the MoCA score as a continuous variable (not shown in this paper), which also found no significant effects of the predictors on the MoCA score. The range of MoCA scores and the age range of our participants were comparable to those examined in the study by Bidelman et al. (2017). However, we would like to point out that the low MoCA group in their study was on average seven years older than the normal group, whereas in our study the difference was only three years, which could also have influenced the results.

Ideally, the inclusion of participants with clinically diagnosed MCI would have increased the robustness of our study. Furthermore, the study would have benefited from including a comprehensive neurocognitive test battery to provide improved cognitive grouping that was not solely based on the MoCA score. Our decision to use the MoCA was influenced by its ease of use and high validity as a rapid clinical screening tool for MCI (Sala et al., 2020), which is consistent with the methods used by Bidelman et al. (2017). In summary, although our study provides valuable insights into the cognitive functioning of older adults, the scope for interpretation is limited by participant selection factors and MoCA-based cognitive grouping.

### Methodological challenges of the subcortical TRF analysis

There are some considerations and limitations of the subcortical models that we would like to point out. To begin with, our TRF models were trained using approximately 17 min of data. While this duration is generally adequate for cortical models (Crosse et al., 2021), its sufficiency for subcortical models is less certain. The main reason for not recording longer was that this paradigm was part of a larger EEG data collection and we were therefore required to keep the audiobook part short. Kulasingham et al. (2024) emphasized that a minimum of 12 min is necessary to achieve a positive SNR in single V-like wave peaks derived from TRF models, but they also showed that with increasing recording length, the SNR improves significantly. This suggests that a longer training period would have yielded more robust subcortical models in our study. In addition, some of our subcortical TRF models performed poorly on the test set, as evidenced by negative encoding accuracies in some participants (Fig. 3). On the one hand, this could be partly due to our methodological decision to optimize the hyperparameter *λ* by leave-one-out cross-validation and train the final model on the entire training set instead of using nested cross-validation (as used by Fuglsang et al., 2020; Bachmann et al., 2021). The rationale was that we did not have enough training data to justify a nested cross-validation approach. On the other hand, our approach might have led to overfitting of the models, which could explain why the models did not generalize well in the test set. Another point of concern is the latency of wave V-like peaks in our subcortical TRF models. Compared to Maddox and Lee (2018), reporting an average peak latency of 6.2 ms in younger participants (19–45 years), our latencies were comparably larger. However, the peak latency was comparable to the latency found by Bachmann (2022), who reported an average peak latency of 8.7 ms in older participants (54–80 years) with hearing loss.

In general, subcortical TRF models are not as well established as their cortical counterpart. We adapted our model fitting approach, which uses linearized ridge regression, to methods commonly used in cortical models (Fuglsang et al., 2020; Bachmann et al., 2021, for example). In contrast, other studies used frequency domain methods for subcortical TRF analysis (Maddox and Lee, 2018; Kulasingham et al., 2024; Shan et al., 2024). In addition, different speech features, such as, e.g., rectified speech waves or firing rates of the auditory nerve, have already been used in subcortical TRF modeling. Determining the most effective method and the minimum data length required for reliable subcortical TRF models is still under investigation and requires further studies, such as those by Kulasingham et al. (2024) and also by Bachmann et al. (2023), who showed that subcortical TRF models can be reliably estimated even when speech is presented in the sound-field, which then requires a slightly longer recording time. The need for longer recordings for accurate subcortical TRF models would question their practical applicability, especially in clinical settings. What does speak in favor of the practical applicability and also for the TRF framework is the fact that the subcortical TRF models can be estimated from the same data as the cortical models. Thus, data acquisition—once the subcortical TRF is established in clinical application—would no longer be associated with the effort of a separate paradigm (such as click or syllable paradigms). Overall, however, the application of subcortical TRF models in clinical practice remains an open question that requires further research. Our study contributes to this ongoing research by applying subcortical TRF models to a specific research question and helping to understand their application and limitations.

### Conclusion and outlook

The search for early markers of cognitive decline remains a central topic in research on aging and cognition. Our study represents a first step in investigating the subcortical and cortical processing of natural continuous speech in cognitive decline for its clinically relevant potential. Our results did not indicate a significant association between early signs of cognitive decline and differences in auditory processing of natural continuous speech. Further research is essential to investigate the components of auditory cognitive speech processing in the context of cognitive decline. In particular, a better understanding of the relationships between different components of auditory processing, such as subcortical and cortical features, hearing loss, and their associations with cognitive decline, remains essential. In addition, exploring other components of auditory processing in terms of their predictive potential is crucial to expand our knowledge. Investigating these components could pave the way for more comprehensive and accurate methods for early detection and monitoring of cognitive decline. This study emphasizes the need to continue the study of auditory cognitive markers, focusing on a more comprehensive assessment of their interaction and predictive abilities, in order to make progress in the early detection and understanding of cognitive decline.

## Synthesis

Reviewing Editor: Anne Keitel, University of Dundee Decisions are customarily a result of the Reviewing Editor and the peer reviewers coming together and discussing their recommendations until a consensus is reached. When revisions are invited, a fact-based synthesis statement explaining their decision and outlining what is needed to prepare a revision will be listed below. The following reviewer(s) agreed to reveal their identity: Marlies Gillis, Fabian Schmidt.

Revise and Re-review

The reviewers and editor agreed that this is an interesting and important study. However, several issues were brought up by the reviewers, many of these will help to make the interpretation of results more robust. These can be summarised as follows:

1) Provide results of analysis when the MoCA scores are used as continuous variable and consider changing this in the manuscript to avoid dichotomisation. Also explore MoCA subscales.

2) Test for evidence for the null hypothesis.

3) Avoid computing effects on average channel data, for example by using cluster based permutation test.

You will find these main issues as well as additional comments in the unabridged reviewer comments below. Please address all comments in a point-by-point manner.

*****

Reviewer #1 - Advances the Field (Required)

This study delves into how mild cognitive impairment impacts auditory processing, using various measures derived from AEP, FFR, or TRF analysis. Surprisingly, the findings reveal no significant alterations in auditory processing associated with the development of cognitive impairment.

The study's limitations lie in the absence of a more challenging cognitive task, like assessing speech in noisy environments, and in dividing participants into two groups based solely on a MoCA score of 26. Nevertheless, the discussion navigates and addresses these limitations.

While the study did not yield statistically significant findings, I believe it's crucial to publish these results to combat publication bias. Although I have no major critiques, there are several minor points and additional questions that arise out of curiosity.

Reviewer #1 - Statistics

Looks done well (although I have no experience with GLMs)

Reviewer #1 - Extended Data

The link to the code was made unavailable, hence, this was not checked.

Reviewer #1 - Software Comments

The link to the code was made unavailable, hence, this was not checked.

Reviewer #1 - Concerns about human or animal subjects

No concerns

Reviewer #1 - Comments to the Authors (Required)

This study delves into how mild cognitive impairment impacts auditory processing, using various measures derived from AEP, FFR, or TRF analysis. Surprisingly, the findings reveal no significant alterations in auditory processing associated with the development of cognitive impairment.

The study's limitations lie in the absence of a more challenging cognitive task, like assessing speech in noisy environments, and in dividing participants into two groups based solely on a MoCA score of 26. Nevertheless, the discussion navigates and addresses these limitations.

While the study did not yield statistically significant findings, I believe it's crucial to publish these results to combat publication bias. Although I have no major critiques, there are several minor points and additional questions that arise out of curiosity.

1) Age and Hearing Capacity Effects:

- Have you observed any variations in AEP/TRF peak latencies based on age or hearing capacity in your study? Gillis et al. showed age-related neural tracking changes. Do you think similar patterns might emerge in your data?

- Considering Gillis et al. used tasks like Stroop and RST to express cognition, do you anticipate different outcomes if a more comprehensive cognitive screening, incorporating tasks like Stroop and RST, were included in your study?

Source: Gillis, M., Kries, J., Vandermosten, M., & Francart, T. (2023). Neural tracking of linguistic and acoustic speech representations decreases with advancing age. NeuroImage, 267, 119841

2) Detailed Analysis of MoCA Scores:

- Have you explored different domains within the MoCA scale to understand if specific domains (e.g., attention or working memory) might better explain the observed effects in your data?

- Could a more comprehensive assessment beyond MoCA provide a clearer understanding of cognitive status, considering MoCA might offer a relatively broad view?

3) MoCA Score as a Continuous Variable and Test Reliability:

- What might be the impact if MoCA scores were considered as a continuous variable rather than solely for group discrimination? The discussion mentioned scores around 26 being ambiguous. Could treating MoCA as a continuous scale might be more insightful?

- Regarding MoCA's reliability, do you have insights into its test-retest reliability? How much do scores vary if the same test is performed after a two-week interval?

MINOR POINTS

L 54-55: You have stated that there is an increased response in both components, while three peaks are present in the AEP. Please better phrase what is meant with "one component", i.e., peaks (which peaks?) or presumed processes (in this case, all 3 peaks)

L 71-72: Another advantage of using the TRF models would be that multiple levels of auditory processing can be tested at once (aka no separate crafted stimuli needed to test the AEP and FFR, but one stimulus can be used to test cortical and subcortical processing)

L 74: please clarify what is meant by dual-recording EEG paradigm so that no additional literature delve is needed into Bidelman et al. and Bachmann 2022 et al. Neither in the stimuli descriptions nor the EEG recording description do I notice a special EEG paradigm...

L 122: How are the participants with moderate hearing loss accommodated? Was the sound intensity increased? Were they included in the analysis? How were the hearing-impaired participants divided into the two groups? From Fig 1., they are mainly in the MoCA normal group; is this correct?

Fig 1: Change the axis (panel A). The last barplot should be on 30 as the MoCA only reaches until 30. (Panel C) Additionally, adding the 34 dB HL as a vertical line might be interesting to denote that this is the cutoff for the hearing-impaired participants.

L 154-191: Regarding the stimulus presentation, I assume the stimulus was presented bilaterally (since symmetric hearing loss was required). Please add this clearly to the manuscript.

L 222: Is adding the ABR of participants using a different electrode valid? Are there better options that would be more valid (such as interpolation of Cz)? By choosing the ABR in another electrode channel, one can presume it measures the response of different neural sources compared to Cz.

L 224: Why have you applied the notch filter on only the harmonics between 50 and 1000 Hz while the harmonics up to the sampling frequency (16384 Hz) might still be in the recording? Have you checked whether all harmonics are sufficiently removed?

L 226: You mention that all additional filters are FIR filters with the given specifications. So, the high pass filter at 80 Hz and the anti-alias filter all have the same band ripple and stopband attenuation? Please clarify the filter section; it is unclear which passband frequencies of the filters. It is unclear what the resulting bandpass filter on the EEG is (I assume it is 80 - 1365Hz; is this correct?)

L 252: Why are the epochs individually high pass filtered at 1 Hz and not for the whole recording? By doing it on the entire recording, filter artifacts can be diminished.

L: 250: It is good to specify how epoch segmentation is done. Is this done by segments of the story (as later inferred from), or was this done differently? How long is such an epoch? Does it also contain data before the start of the segment?

L 267: Is it correct that you have 25 epochs (as you have 25 segments) to calculate the AEP?

L 283: Why do you nan-pad while not truncating to the shortest segment? Does this mean all segments are nan-padded until 48 s (since one segment is, on average, 45s)?

L 287: Writing mistake: whith - with?

L 305: A final model was trained on the training set with optimal lambda: why not leave out cross-validation and resulting TRF and prediction accuracy averaged across folds? If only one final model is used, which folders are selected as training and testing sets? It is unclear whether the unseen test set is also unseen for the lamba determination. Please clarify how models were estimated.

L 309: Without knowing the prior research, this section is rather unclear; please add additional information. Why do you fit and average the subcortical model for the original and invested speech features? A short extra motivation might clarify these sentences.

*****

Reviewer #2 - Advances the Field (Required)

The authors investigate the role of neural speech processing in relation to cognitive decline. Cognitive decline has been related to hearing ability in the past and is considered to be one of the best predictors of dementia (Armstrong et al. 2019; Livingston et al., 2020).

This makes the investigation of auditory processing in cognitive decline, above and beyond pure tone hearing ability, an exciting, important and interesting research topic. The manuscript is motivated by the auditory hypersensitivity theory, which claims that auditory responses in (sub-)cortical areas are enhanced in cognitive decline. Crucially, the authors do neither find support for this theory with regards to the processing of continuous speech nor for the processing of simpler acoustic sounds (clicks or acoustic speech onsets).

The manuscript is well written, employs novel and well-documented methods and discusses the presented results as well as their limitations thoroughly. Furthermore, finding and reporting no evidence for something is (IMHO) just as important as finding evidence for something.

Armstrong, N. M., An, Y., Doshi, J., Erus, G., Ferrucci, L., Davatzikos, C., ... & Resnick, S. M. (2019). Association of midlife hearing impairment with late-life temporal lobe volume loss. JAMA otolaryngology-head & neck surgery, 145(9), 794-802.

Livingston, G., Huntley, J., Sommerlad, A., Ames, D., Ballard, C., Banerjee, S., ... & Mukadam, N. (2020). Dementia prevention, intervention, and care: 2020 report of the Lancet Commission. The Lancet, 396(10248), 413-446.

Reviewer #2 - Statistics

The statistical analysis appears to be correct, but could be extended to better support the claims of the authors.

Reviewer #2 - Comments to the Authors (Required)

General

The authors investigate the role of neural speech processing in relation to cognitive decline. Cognitive decline has been related to hearing ability in the past and is considered to be one of the best predictors of dementia (Armstrong et al. 2019; Livingston et al., 2020).

This makes the investigation of auditory processing in cognitive decline, above and beyond pure tone hearing ability, an exciting, important and interesting research topic. The manuscript is motivated by the auditory hypersensitivity theory, which claims that auditory responses in (sub-)cortical areas are enhanced in cognitive decline. Crucially, the authors do neither find support for this theory with regards to the processing of continuous speech nor for the processing of simpler acoustic sounds (clicks or acoustic speech onsets).

The manuscript is well written, employs novel and well-documented methods and discusses the presented results as well as their limitations thoroughly. Furthermore, finding and reporting no evidence for something is (IMHO) just as important as finding evidence for something.

This brings me also to my first inquiry. Presently, the results of the authors show the absence of evidence for neural speech processing in relation to cognitive decline. That is they didn't observe any statistically significant differences between the normal and low MoCA groups. I think the authors could increase the impact of their manuscript, by additionally actually testing for the evidence of absence of neural speech processing in relation to cognitive decline.

There are several ways/metrics both utilizing either frequentist or bayesian methodology to further explore this. Testing this is IMHO not necessary, but could deliver interesting insights and allow the authors to make stronger claims about their results.

Frequentist:

Two one-sided tests against a region of practical equivalence (TOST)

https://journals.sagepub.com/doi/10.1177/2515245918770963

Bayesian:

Bayesian Parameter estimation using a region of practical equivalence

https://journals.sagepub.com/doi/full/10.1177/2515245918771304

Bayes Factors

https://psycnet.apa.org/record/2022-00801-001

Furthermore, in the current version of the manuscript the authors compare several features of the grand average TRF across channels (e.g. N1-P2 differences) between MoCA groups. Computing effects on grand averages ignores potential effects on clusters of electrodes that might disappear/cancel in averaging. I would recommend that the authors rerun their analysis on "channel level" correcting for multiple comparisons (e.g. via cluster based permutation tests; Maris & Oostenveld, 2007) to find out whether or not such clusters exist. I think that running this analysis would significantly increase the value of the paper.

Minor:

In their discussion the authors take a critical stand on their approach to separate participants in two groups based on their MoCA score. I can understand that this initial decision was based on trying to replicate the findings by Bidelman et al. 2017 that used a similar cut-off (MoCA = 26). I am in general not the biggest fan of discretizing continuous variables as valuable information is lost in the process. So I was wondering whether it wouldn't be more informative to keep the MoCA scale continuous and rerun the analysis with an appropriate GLM. However, I don't really consider doing this analysis to be strictly necessary for publication and more as an interesting additional information.

TRF Models:

Using acoustic material processed via biophysical models of the auditory pathway as regressors is a clever approach (e.g. Shan et al. 2024) that may be useful in revealing intricacies of auditory processing. This approach has also been used to model brain activity (eg. Lindboom et al. 2023). Given both the availability of these tools as well as the authors ability to properly use them I was left wondering why they were only applied to model subcortical activity? Doing so is IMHO neither mandatory nor necessary for publication, but it may be an interesting venue to explore in future analyses.

Statistical Analysis:

To test their main hypothesis the authors use a GLM with a logit-link and assume a Bernoulli distribution for their dependent variable. As far as I know this is equivalent to a logistic regression. It might be easier and more descriptive to refer to this also as such throughout the manuscript.

Figure 3:

In Figure 3 the authors show that there is no within group correlation for low/high MoCA and encoding accuracy at cortical and subcortical level. It is not entirely clear to me what point the authors are trying to make with this, as this result is also not further discussed in the manuscript (apologies if I missed the point in the results/discussion). In the same paragraph in which Figure 3 is displayed (5.2 Encoding accuracies) the authors state that people with a "Low" and "Normal" MoCA score don't differ significantly in their (sub-)cortical encoding accuracies. While this information can be extracted from Figure 3, I think it would be more straightforward to show this more directly e.g. as done in Figure 4a or 5a using a point/violin plot.

References:

Armstrong, N. M., An, Y., Doshi, J., Erus, G., Ferrucci, L., Davatzikos, C., ... & Resnick, S. M. (2019). Association of midlife hearing impairment with late-life temporal lobe volume loss. JAMA otolaryngology-head & neck surgery, 145(9), 794-802.

Maris, E., & Oostenveld, R. (2007). Nonparametric statistical testing of EEG-and MEG-data. Journal of neuroscience methods, 164(1), 177-190.

Lindboom, E., Nidiffer, A., Carney, L. H., & Lalor, E. C. (2023). Incorporating models of subcortical processing improves the ability to predict EEG responses to natural speech. Hearing Research, 433, 108767.

Livingston, G., Huntley, J., Sommerlad, A., Ames, D., Ballard, C., Banerjee, S., ... & Mukadam, N. (2020). Dementia prevention, intervention, and care: 2020 report of the Lancet Commission. The Lancet, 396(10248), 413-446.

Shan, T., Cappelloni, M. S., & Maddox, R. K. (2024). Subcortical responses to music and speech are alike while cortical responses diverge. Scientific Reports, 14(1), 789.
